# Discovery of a cystathionine *γ*-lyase (CSE) selective inhibitor targeting active-site pyridoxal 5′-phosphate (PLP) via Schiff base formation

**DOI:** 10.1038/s41598-023-43536-6

**Published:** 2023-09-30

**Authors:** Honami Echizen, Kenjiro Hanaoka, Kazuhito Shimamoto, Ryota Hibi, Sachiko Toma-Fukai, Hisashi Ohno, Eita Sasaki, Toru Komatsu, Tasuku Ueno, Yukihiro Tsuchiya, Yasuo Watanabe, Takao Otsuka, Hiroaki Saito, Satoru Nagatoishi, Kouhei Tsumoto, Hirotatsu Kojima, Takayoshi Okabe, Toshiyuki Shimizu, Yasuteru Urano

**Affiliations:** 1 Graduate School of Pharmaceutical Sciences, The University of Tokyo, 7-3-1 Hongo, Bunkyo-ku, Tokyo, 113-0033 Japan; 2 Graduate School of Pharmaceutical Sciences, Keio University, 1-5-30 Shibakoen, Minato-ku, Tokyo, 105-8512 Japan; 3 Graduate School of Science and Technology, Nara Institute of Science and Technology, Nara, 630-0192 Japan; 4 Faculty of Pharmaceutical Sciences, Showa Pharmaceutical University, Machida-shi 194-8543, Tokyo, Japan; 5 Graduate School of Medicine, Kyoto University, 53 Shogoin-Kawaharacho, Sakyo-ku, Kyoto, 606-8507 Japan; 6 Faculty of Pharmaceutical Sciences, Hokuriku University, 3 Ho Kanakawa-cho, Kanazawa, Ishikawa 920-1181 Japan; 7 Medical Device Development and Regulation Research Center, School of Engineering, The University of Tokyo, 7-3-1 Hongo, Bunkyo-ku, Tokyo, 113-8656 Japan; 8 Department of Bioengineering, School of Engineering, The University of Tokyo, 7-3-1 Hongo, Bunkyo-ku, Tokyo, 113-8656 Japan; 9 Drug Discovery Initiative, The University of Tokyo, 7-3-1 Hongo, Bunkyo-ku, Tokyo, 113-0033 Japan; 10 Graduate School of Medicine, The University of Tokyo, 7-3-1 Hongo, Bunkyo-ku, Tokyo, 113-0033 Japan

**Keywords:** Chemical libraries, Chemical tools, Enzyme mechanisms, Drug screening

## Abstract

D,L-Propargylglycine (PAG) has been widely used as a selective inhibitor to investigate the biological functions of cystathionine *γ*-lyase (CSE), which catalyzes the formation of reactive sulfur species (RSS). However, PAG also inhibits other PLP (pyridoxal-5′-phosphate)-dependent enzymes such as methionine *γ*-lyase (MGL) and L-alanine transaminase (ALT), so highly selective CSE inhibitors are still required. Here, we performed high-throughput screening (HTS) of a large chemical library and identified oxamic hydrazide **1** as a potent inhibitor of CSE (IC_50_ = 13 ± 1 μM (mean ± S.E.)) with high selectivity over other PLP-dependent enzymes and RSS-generating enzymes. Inhibitor **1** inhibited the enzymatic activity of human CSE in living cells, indicating that it is sufficiently membrane-permeable. X-Ray crystal structure analysis of the complex of rat CSE (rCSE) with **1** revealed that **1** forms a Schiff base linkage with the cofactor PLP in the active site of rCSE. PLP in the active site may be a promising target for development of selective inhibitors of PLP-dependent enzymes, including RSS-generating enzymes such as cystathionine *β*-synthase (CBS) and cysteinyl-tRNA synthetase 2 (CARS2), which have unique substrate binding pocket structures.

## Introduction

Reactive sulfur species (RSS) have attracted increasing interest in the past two decades as their multiple roles in biological systems have become apparent. For example, hydrogen sulfide (H_2_S) is a gaseous signaling molecule that is involved in vasodilation, inflammation, and angiogenesis, and also exhibits antioxidant activity^1–4^. More recently, many researchers have focused on sulfane sulfur (S^0^)^5^, which binds reversibly to other sulfur atoms in biological molecules such as reduced glutathione (GSH) and cysteine, forming persulfides (–S–SH) and polysulfides (–S–S_n_–S–). Sulfane sulfur plays an important role in signal transduction and redox regulation^6^. So far, four RSS-generating enzymes have been identified: cystathionine *γ*-lyase (CSE), cystathionine *β*-synthase (CBS), 3-mercaptopyruvate sulfurtransferase (3MST), and cysteinyl t-RNA synthetases (CARSs)^7–9^.

CSE produces H_2_S during the metabolism of cysteine, and also generates cysteine persulfide (CysSSH) from cystine as a substrate^10^. Nevertheless, the biological functions of CSE in terms of RSS production and/or metabolism remain to be fully elucidated. D,L-Propargylglycine (PAG) has been widely used for this purpose as an selective CSE inhibitor, but it also inhibits other PLP-dependent enzymes such as methionine *γ*-lyase (MGL) and L-alanine transaminase (ALT)^11–14^. Therefore, there is a need for potent and specific CSE inhibitors as research tools.

In this study, we did not employ a molecular design approach to develop a selective CSE inhibitor, but rather adopted a high-throughput screening (HTS) approach, using our fluorescence probe to evaluate a library of 161,600 compounds. We successfully identified a potent and highly selective CSE inhibitor that works in live cells, and also established its inhibitory mechanism. We believe our findings open up a novel design strategy for selective inhibitors of PLP-dependent enzymes by targeting PLP itself.

## Results

### HTS of a large chemical library to identify CSE inhibitors

**Figure 1:**
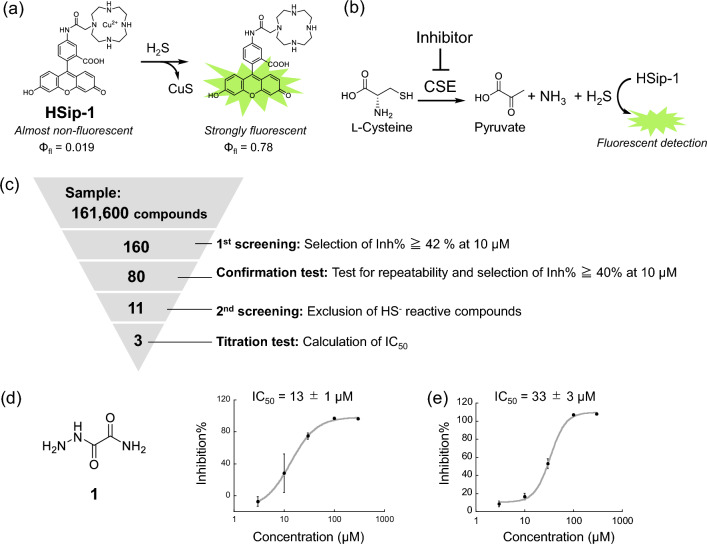
(**a**) Structure, fluorescence quantum yields and detection mechanism of HSip-1, an H_2_S-detecting fluorescence probe that utilizes the complexation of HS^−^ with Cu^2+^. (**b**) Detection mechanism of the enzymatic activity of CSE for inhibitor screening with HSip-1. (**c**) The result of HTS for CSE inhibitors from a chemical library of 161,600 compounds. (**d**) Structure of hit compound **1**, and its inhibitory activity towards rCSE (mean ± S.E.) determined by fluorescence assay with HSip-1. Error bars represent ± S.D. (n = 4). (**e**) Inhibitory activity of **1** toward the production of sulfane sulfur (S^0^)-containing cysteine persulfide by rCSE (mean ± S.E.). Sulfane sulfur was detected with the fluorescence probe SSP4 (Dojindo Laboratory). Error bars represent ± S.D. (n = 4).

We have previously developed a fluorescence probe for H_2_S, HSip-1 (Fig. 1a)^15^, which has a fluorescein (fluorophore) scaffold and an azamacrocyclic Cu^2+^ complex moiety linked by an amide bond. HSip-1 is almost non-fluorescent before reaction with H_2_S, but becomes strongly fluorescent in aqueous solutions upon addition of H_2_S. We previously utilized this fluorescence probe in the HTS of 174,118 compounds, resulting in the discovery of a selective 3MST inhibitor^16^. Here, we aimed to establish a fluorescence-based HTS system to find CSE inhibitors, using HSip-1 for the detection of CSE enzymatic activity.

The developed assay system employs a plate reader to detect H_2_S production by CSE as a fluorescence signal in a solution containing 30 μg/mL rat CSE (rCSE), 1 μM HSip-1, 100 μM pyridoxal-5′-phosphate (PLP) and 1.5 mM cysteine (a substrate) in a 384-well plate format (Fig. 1b). A large amount of rCSE was expressed in *E. coli* and purified on a Ni Sepharose 6 Fast Flow module. We then performed screening of a chemical library consisting of 161,600 compounds (from the Drug Discovery Initiative, the University of Tokyo) for CSE inhibitors (Fig. 1c). In the 1st screening, the inhibitory activity of all compounds was assessed at the concentration of 10 µM (n = 1), and 160 compounds that showed more than 42% inhibition were selected. Then, a confirmation test for repeatability (n = 4) of the 160 compounds selected in the 1st screening was performed to exclude false-positives. In this step, we also excluded cysteine-reactive compounds by changing the addition order of the rCSE and cysteine (substrate) solutions. In other words, in the 1st screening, the enzyme solution was firstly added to each library compound, while in the confirmation test for repeatability of the hit compounds in the 1st screening, we firstly added the cysteine solution to each compound. This enabled us to identify and exclude cysteine-reactive compounds, based on their reaction with the added cysteine. These procedures left 80 compounds showing both more than 40% inhibition at 10 µM and similar % inhibition of rCSE irrespective of the addition order of the rCSE and cysteine solutions. In the 2nd screening, we examined the reactivity of these 80 compounds with H_2_S using an H_2_S donor^17^ to exclude H_2_S-reactive compounds. In this assay, we employed 1 µM HSip-1, 50 µM H_2_S donor, a selected compound (0.25, 1.0, 3.0, 10 and 30 μM) and 1 mM cysteine without rCSE in 30 mM HEPES buffer (pH 7.4); under these conditions, if a compound directly reacts with H_2_S, HSip-1 does not show a fluorescence increase. In this assay, we selected 13 compounds which were non-reactive to H_2_S, but one was relatively unstable and two of them were the same compound, so that 11 compounds were finally selected. These compounds included some hydrazine and hydrazide derivatives, so we performed titration assay of the selected 11 compounds together with 9 commercially available hydrazine and hydrazide compounds (Supplementary Fig. S3) and calculated the half-maximal (50%) inhibitory concentration (IC_50_) of each compound. We also resynthesized some selected compounds and examined their IC_50_ values to verify that the selected compounds actually inhibit rCSE. Indeed, some of the resynthesized compounds did not inhibit rCSE. Finally, based on these assessments, we chose oxamic hydrazide (**1**) as a promising CSE inhibitor (IC_50_ = 13 ± 1 µM) (Fig. 1d).

### Assessment of rCSE-inhibitory ability of inhibitor 1

Next, in order to further confirm the CSE-inhibitory ability of **1**, we employed a gas chromatographic method. A mixture of 30 μg/mL rCSE, 100 μM PLP and 1.5 mM cysteine in 30 mM HEPES buffer (pH 7.4) with or without **1** or PAG was incubated in a sealed glass vial, and the air containing enzymatically produced H_2_S in the glass vial was analyzed by gas chromatography. The inhibitory activity of **1** was confirmed, and the IC_50_ evaluated by this method was 23 ± 6 µM (Supplementary Fig. S4), in good agreement with the result of fluorescence assay.

We further examined whether or not** 1** can inhibit the production of cysteine persulfide from cystine by CSE (Supplementary Fig. S5)^10^, using a commercially available sulfane sulfur-detecting probe, SSP4. As expected, cysteine persulfide production by CSE was inhibited by **1** with an IC_50_ value of 33 ± 3 µM (Fig. 1e). This result suggests that H_2_S and cysteine persulfide are produced at the same active site in CSE.

**Figure 2:**
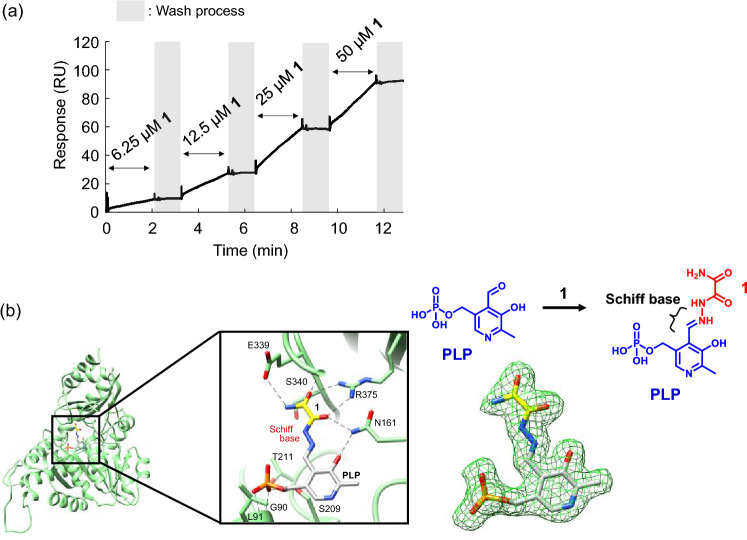
(**a**) SPR measurement of binding between rCSE and** 1**. Various concentrations of **1** (6.25, 12.5, 25, 50 µM) in the buffer solution were flowed on an SPR sensor chip. Gray regions in the figure indicate washing of the sensor chip with the buffer solution not containing **1**. (**b**) X-Ray cocrystal structure of rCSE and **1**. PLP and **1** form a Schiff base linkage in the protein. The Fo-Fc electron density map (green) of the complex of **1** and PLP is shown at level 0.234 (nearly equal to the 3 sigma level).

Moreover, we performed surface plasmon resonance (SPR) assay to investigate the binding kinetics between rCSE and **1**. rCSE was immobilized on a sensor chip of an SPR sensor, and various concentrations (6.25, 12.5, 25 and 50 µM) of **1** were flowed over it. The sensor chip was flushed with buffer solution not containing **1** before each concentration change of **1**. The binding of **1** to rCSE increased concentration-dependently (Fig. 2a). In SPR binding studies between small molecule ligands and proteins, the washing process often markedly decreases the SPR signal. Interestingly, however, **1** showed a relatively small signal decrease, suggesting that little dissociation occurs during the washing process. Therefore, our SPR data suggests that the interaction between rCSE and **1** is strong.

**Figure 3:**
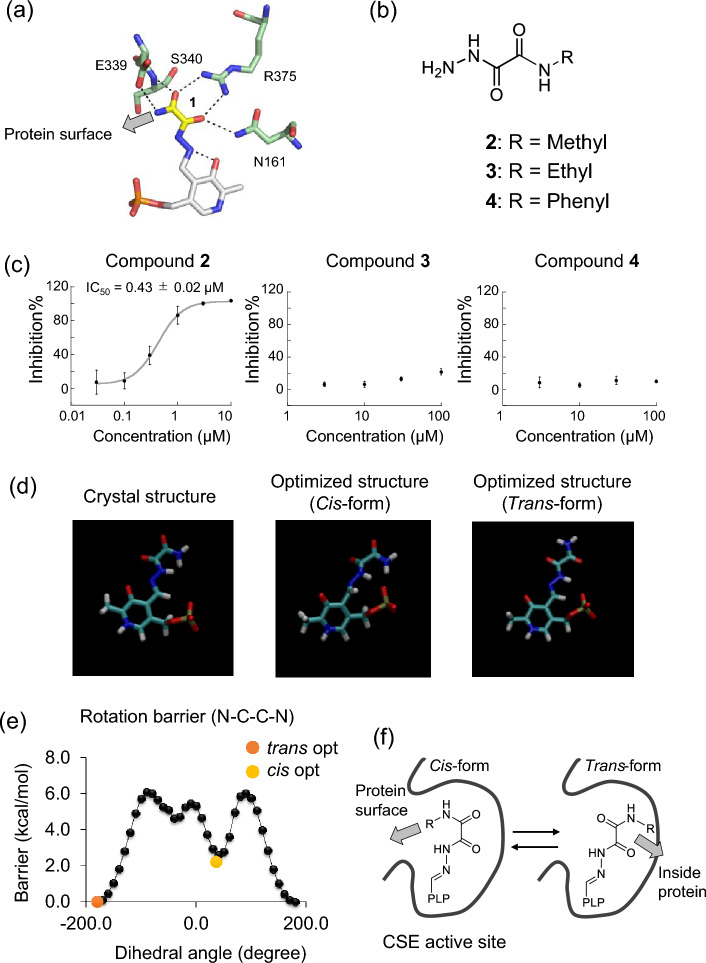
(**a**) Interactions between amino acid residues of rCSE and **1** conjugated with PLP at the active site in the cocrystal structure of rCSE and **1**. (**b**,**c**) Structures of synthesized derivatives of **1** and their rCSE-inhibitory activity (mean ± S.E.) assessed by fluorescent detection with HSip-1. Error bars represent ± S.D. (n = 4). (**d**) Structure of the complex of **1** and PLP in the cocrystal structure of rCSE and **1** (left), optimized stable conformation of the complex in the *cis* conformation (middle), and optimized stable conformation of the complex in the *trans* conformation (right). (**e**) The conformational energy profile of **1** as a function of dihedral angle (N–C–C–N). Each geometry was optimized at the HF/6-31G*/PCM(water) level. The structural energies of the optimized *cis* and *trans* conformers of **1** are also plotted. (**f**) Schematic illustration of the complex of **1** and PLP in *cis* or *trans* form in the active site of CSE.

We then determined the high-resolution (1.9 Å) X-ray crystal structure of the rCSE complex with **1**, and found that the hydrazide group of **1** forms a Schiff base linkage with the aldehyde group of PLP in the active site of rCSE, affording PLP-**1** (Fig. 2b, Supplementary Fig. S6). Moreover, several amino acid residues of rCSE (Glu^339^, Ser^340^, Arg^375^ and Asn^161^) around the CSE active site form hydrogen bonds with oxygen atoms of **1** in PLP-**1** (Fig. 3a). These results are consistent with the observed strong binding of **1** to rCSE in the SPR measurement.

### Design, synthesis and evaluation of derivatives of 1

Next, we designed and synthesized several derivatives of **1** to explore the structure–activity relationship. We focused on the terminal amino group of PLP-**1**, which faces the protein surface of rCSE (Fig. 3a), and synthesized three derivatives (compounds **2**–**4)** with simple modifications of the terminal amino group (Fig. 3b and Supplementary Information for synthetic details). In in vitro fluorescence assay with HSip-1, **2** showed more potent inhibitory ability (IC_50_ = 0.43 ± 0.02 µM) than **1**, whereas **3** and **4** showed almost no inhibitory activity towards rCSE (IC_50_ > 100 µM) (Fig. 3c).

To investigate the reason for this result, we performed calculations focusing on the conformation of the dicarbonyl structure of PLP-**1**. We first optimized the structures of the *cis* and *trans* forms of PLP-**1** by quantum chemical (QC) calculation. The optimized *cis* form showed a dihedral angle defined by N–C–C–N of the dicarbonyl structure of PLP-**1** of around 40 degrees, while the energy minimum of the *trans* form was located near the dihedral angle of 180 degrees (Fig. 3d). These results indicate that PLP-**1** can take both *cis* and *trans* conformations, though the *cis*-form of PLP-**1** appears to be preferred in the crystal structure as judged from B-factor analysis (Fig. 3a and Supplementary Fig. S7). We then performed QC calculation of **1** by changing the dihedral angle of N–C–C–N. The results show that the *trans*-form of **1** is more stable than the *cis*-form, and there is an energy barrier of about 4 kcal/mole between them (Fig. 3e).

We next examined the interconvertibility of the *cis* and *trans* forms in rCSE by molecular dynamics (MD) calculation using the cocrystal structure of rCSE and **1** determined by the X-ray crystal analysis, as shown in Fig. 2b. We first performed structural optimization of PLP-**1** in the *cis* form, and found that it could stably interact with Asn^161^, Glu^339^, Ser^340^ and Arg^375^ of rCSE. Then, the dihedral angle analysis was performed to evaluate the stability of PLP-**1** in the *cis* form when the temperature was changed from 10 to 300 K (Supplementary Fig. S8). The MD calculations indicated that PLP-**1** would take the *cis* form at less than 200 K, but the conformation changed to the *trans* form as the temperature was increased (Supplementary Fig. S8 and Supplementary Movie S1 (MD calculation at 240 K)). These results suggested that PLP-**1** can take the *trans*-conformation at room temperature as well as the *cis*-conformation. The terminal amino group of PLP-**1** in rCSE would face toward the outside of the protein (the solvent side) in the *cis*-conformation, whereas in the *trans*-conformation it would face the inside of the protein, as shown in Fig. 3f. Therefore, the ability of derivatives** 3** and **4**, which have bulky substituents, to take the *trans* conformation as well as the *cis* conformation might account at least in part for their relatively weak inhibitory activities.

### Assessment of selectivity of inhibitor 1 for CSE

**Figure 4:**
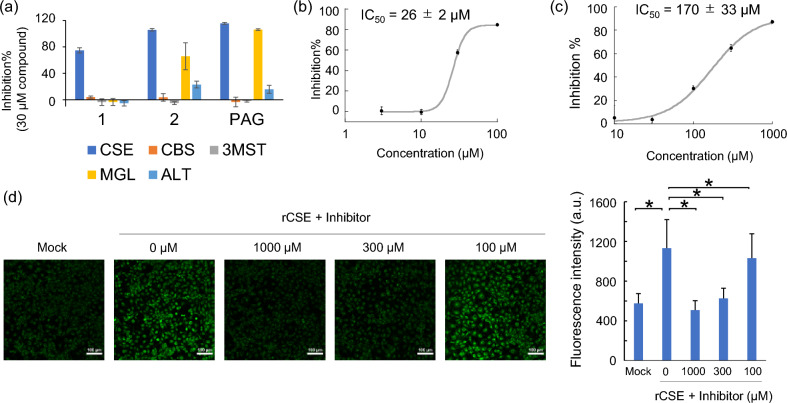
(**a**) Inhibitory activity at 30 µM **1**, **2** and PAG towards RSS-generating enzymes (rCSE, rCBS and m3MST) and other PLP-dependent enzymes (MGL and ALT). The fluorescence intensity (or absorbance) of the assay mixtures without inhibitor was defined as 0% inhibition, and that of the assay mixtures without enzyme was defined as 100% inhibition. (**b**) Inhibitory activity of** 1** towards hCSE in cell lysate (mean ± S.E.). H_2_S production in lysate of HEK293T cells expressing hCSE was detected with HSip-1. The cell lysate in HEPES buffer (pH 7.4) was incubated with 1.5 mM cysteine and 100 μM PLP. Error bars represent ± S.D. (n = 4). (**c**) Inhibitory activity of **1** towards hCSE in living cells (mean ± S.E.). HEK293T cells expressing hCSE were pretreated with various concentrations of **1**, and then lysed. The fluorescence intensity was measured after incubation of 450 µg/mL lysate with 100 µM PLP, 1 µM HSip-1, 0.005% tween20 and 1.5 mM cysteine in 20 mM HEPES (pH 7.4). Error bars represent ± S.D. (n = 4). (**d**) Live-cell fluorescence imaging with SSP-4 of sulfane sulfur produced in rCES-expressing COS-7 cells. The cells were treated with inhibitor **1** at various concentrations (0, 100, 300 and 1000 µM) and fluorescence images were captured using a confocal laser scanning microscope with a 20 × objective lens. The excitation and emission wavelengths for SSP4 were 488 nm and 500–550 nm, respectively. *indicates *p* < 0.05 (Student’s *t* test).

The selectivity of **1**,** 2** and PAG for rCSE over other RSS-producing enzymes, CBS and 3MST, was examined (Fig. 4a). CBS is a PLP-dependent enzyme, like CSE, and produces H_2_S from cysteine and homocysteine as substrates. 3MST is also an H_2_S-producing enzyme, but is not PLP-dependent and uses 3-mercaptopyruvate as a substrate. The amount of H_2_S produced by CBS or 3MST was evaluated in the presence or absence of the inhibitor by measuring the fluorescence intensity of HSip-1. All the compounds showed high selectivity for CSE over CBS and 3MST at the concentration of 30 µM (Fig. 4a). We also examined the inhibitory activity towards two other PLP-dependent enzymes, MGL and ALT. MGL metabolizes L-methionine to α-ketobutyric acid, methanethiol and ammonia, and is inhibited by PAG^14^. MGL also produces H_2_S from homocysteine as a substrate, and this activity of MGL can be measured by fluorescence detection with HSip-1. ALT produces pyruvate and L-glutamate from α-ketoglutarate and L-alanine, and is also inhibited by PAG^12^. The enzymatic activity of ALT can be assessed by means of a coupled assay using the reduction of pyruvate to L-lactate with lactate dehydrogenase and NADH and measuring the change in absorption of NADH. We found that **1** showed high selectivity for CSE over MGL and ALT (Fig. 4a). Compound **2** also showed high selectivity for CSE at 3 µM (Supplementary Fig. S9), but showed some inhibitory activity towards MGL and ALT at 30 µM (Fig. 4a). PAG inhibited both MGL and ALT at 30 µM, as previously mentioned. Thus, **1** showed the highest selectivity for CSE among the compounds examined, and so we next investigated its suitability for live-cell experiments.

### Assessment of inhibitory activity of 1 in cell cultures

We first examined whether or not **1** inhibits human CSE (hCSE) in cell lysates. We prepared the lysate of HEK293T cells expressing hCSE, and added 1.5 mM cysteine, 100 μM PLP, 1 μM HSip-1 and various concentrations of **1** to it. Based on fluorescence measurements, the IC_50_ value of **1** for hCSE was calculated to be 26 ± 2 µM, which is similar to the value for rCSE measured in HEPES buffer (Fig. 4b). This result indicates that **1** can inhibit hCSE in cell lysate, as well as rCSE. We also confirmed that all amino acid residues of rCSE involved in the recognition of **1** are conserved in hCSE (Supplementary Fig. S10).

We next examined the suitability of **1** for live-cell experiments. First, we performed CCK-8 assay (Dojindo) to examine its cytotoxicity. COS7 cells were incubated with various concentrations of **1** for 27 h at 37 °C under 5% CO_2_ and the cell viability was assessed. No cytotoxicity was observed below 100 µM (Supplementary Fig. S11). We then examined whether **1** can inhibit the enzymatic activity of hCSE in living cells by employing an indirect approach (Supplementary Fig. S12). HEK293T cells expressing hCSE were incubated with 0–1 mM **1** for 30 min, then washed twice with phosphate-buffered saline (PBS), and lysed in RIPA buffer (50 mM Tris–HCl buffer (pH 7.6), 150 mM NaCl, 1% Nonidet P40 Substitute, 0.5% sodium deoxycholate, protease inhibitor cocktail, 0.1% SDS). The inhibitory activity of **1** was evaluated by fluorescence detection after adding 1.5 mM cysteine, 100 μM PLP and 1 μM HSip-1 to these cell lysates. Compound **1** showed an IC_50_ value of 170 ± 33 μM under these conditions (Fig. 4c), indicating that it is sufficiently cell-membrane permeable and would be available for live-cell applications.

Finally, we evaluated the inhibitory activity of **1** towards rCSE in cells. It is reported that an increased amount of sulfane sulfur (S^0^) was observed in the CSE-overexpressing cells by imaging with the fluorescence probe SSP4^10^. So, we prepared COS-7 cells expressing rCSE, treated them with **1** at various concentrations (0, 100, 300 and 1000 µM), and performed fluorescence imaging of sulfane sulfur with SSP4. The fluorescence intensity was almost completely suppressed by 1000 µM **1** (Fig. 4d). This result is consistent with the inhibitory activity of **1** shown in Fig. 4c. Thus, this inhibitor is available for live-cell experiments.

## Discussion

**Figure 5:**
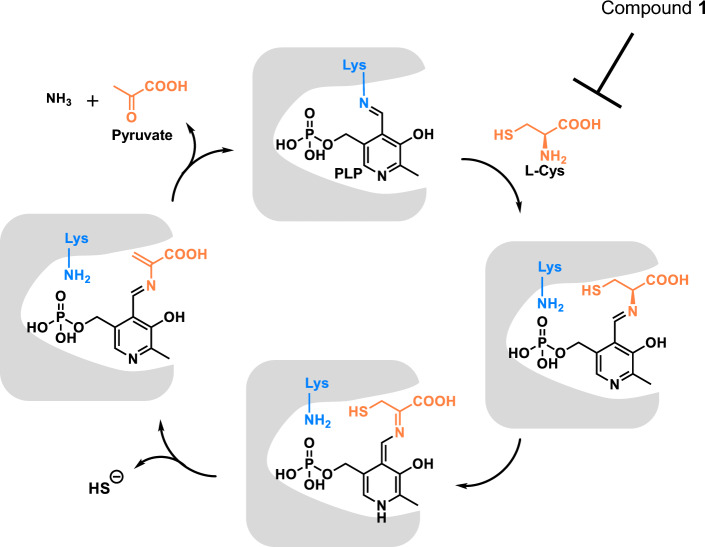
Catalytic mechanism of CSE and the step inhibited by **1**.

Inhibitor screening of 161,600 compounds with our H_2_S-detecting fluorescence probe led to the identification of **1** as a new CSE-selective inhibitor. Notably, the complex of **1** with rCSE showed no dissociation in SPR measurement, and this appears to be due to Schiff base formation of **1** with PLP, a coenzyme in the active site of rCSE. Interestingly, although other H_2_S-producing enzyme CBS is also PLP-dependent, **1** showed high selectivity for CSE over CBS, as well as other PLP-dependent enzymes such as MGL and ALT, which are inhibited by the conventional CSE inhibitor PAG. We think that the reason why **1** shows high selectivity for CSE is that amino acid residues in the active site of CSE play important roles in Schiff base formation between **1** and PLP by interacting with **1** (Fig. 5). The Schiff base formation may be promoted by the proximity effect^18^, considering that **1** and PLP are located close to each other in the binding pocket of CSE.

PLP is an active form of vitamin B6 and acts as a cofactor for more than 140 distinct enzymatic activities^19^. These PLP-dependent enzymes catalyze a diverse range of reactions, including racemization, decarboxylation, *β*-addition, *β*-elimination, retro-aldol cleavage, transamination, and α-elimination. In this context, it is noteworthy that the substrate pocket of rCSE is relatively small in our X-ray crystal structure. For example, the UCSF Chimera program gave a size of 432 Å^3^ for CSE, whereas the substrate pockets of the drug-metabolizing enzymes CYP3A4 and CYP2C9 are about 500–600 Å^3^ in size^20^ (Supplementary Fig. S13). In the pocket of rCSE, PLP occupies 181 Å^3^, while the size of **1** is 71 Å^3^. In addition, PLP is located at a position that restricts entry to the pocket, and it is estimated that the space through which molecules can enter is only about 70 Å^3^. Thus, the covalent bond formation between **1** and PLP seems likely to contribute to the high selectivity and potent inhibitory activity of **1** toward CSE, in addition to the multiple hydrogen bonding interactions between the inhibitor and the amino acid residues at the active site of CSE. Overall, our findings suggest that it may be possible to develop selective inhibitors for various PLP-dependent enzymes by taking into account their active site structure and the location of PLP, thereby opening up a potential new avenue for drug discovery.

In studies of the functions of enzymes, the use of inhibitors and genetic engineering approaches are complementary. For example, the off-target effects of inhibitors can be clarified by knockout of a target protein. Conversely, when a target protein is genetically knocked out, a compensatory mechanism(s) may be activated, altering cell metabolism. The use of inhibitors may allow the effect of inhibiting a target protein to be examined before such compensatory mechanisms can come into effect^21^, and therefore both methods have a place in biological studies.

In summary, we successfully developed a highly selective CSE inhibitor by employing a fluorescence-based HTS method with our fluorescence probe and demonstrated the utility of the developed inhibitor for live-cell experiments. X-Ray crystal analysis indicated that the CSE inhibitor binds covalently to PLP at the active site of CSE. Nevertheless, this inhibitor was highly selective for CSE over other PLP-dependent enzymes such as CBS, MGL and ALT. This result suggests that the PLP-targeting strategy might also be applicable to the development of selective inhibitors for other PLP-dependent enzymes. We believe the inhibitor reported here will be a useful tool not only for elucidating the precise role of CSE in RSS biology, but also for drug discovery.

## Methods

### General materials and instruments

General chemicals were of the best grade available, supplied by Tokyo Chemical Industries, Fujifilm Wako Pure Chemical, Aldrich Chemical Co., Kanto Chemical Co., Dojindo and Invitrogen Corp., and were used without further purification. All solvents were used after appropriate distillation or purification. SSP4 was purchased from Dojindo Laboratories (Kumamoto, Japan). BL21 Competent E. coli was obtained from TAKARA BIO inc. COS7 cells were obtained from ATCC. NMR spectra were recorded on a JEOL JNM-LA300 instrument at 300 MHz for ^1^H NMR and at 75 MHz for ^13^C NMR or a JEOL JNM-AL400 instrument at 400 MHz for ^1^H NMR and at 100 MHz for ^13^C NMR. Mass spectra (ESI^+^ or ESI^−^) were measured with a JEOL JMS-T100LC AccuTOF for ESI. HPLC analyses were performed on an Inertsil ODS-3 (4.6 × 250 mm) column (GL Sciences Inc.) using a HPLC system composed of a pump (PU-2080, JASCO) and a detector (MD-2018 or FP-2025, JASCO). UPLC-MS analyses were performed on a BEH C18 1.7 µm (2.1 × 50 mm) column (ACQUITY UPLC®) using a UPLC system composed of a pump (Acquity UPLC H class, Waters), and a detector (Acquity UPLC and Acquity QDa, Waters). Preparative HPLC was performed on an Inertsil ODS-3 (10 × 250 mm) column (GL Sciences Inc.) using a HPLC system composed of a pump (PU-2080, JASCO) and a detector (MD-2015 or FP-2025, JASCO) or a HPLC system composed of a pump (PU-2086, JASCO) and a detector (MD-2018, JASCO). pH was measured with a F-52 pH meter (Horiba).

### Surface plasmon resonance (SPR)

The interaction between rCSE and oxamic hydrazide was analyzed using a Biacore T200 SPR instrument (Cytiva). A CM7 Biacore sensor chip (Cytiva) was used to immobilize rCSE at approximately 35,000 RU after activation with *N*-hydroxysuccinimide/*N*-ethyl-*N*′-(3-dimethylaminopropyl) carbodiimide hydrochloride. Acetate buffer (pH 5.5) was used to dilute rCSE. The activated surface of the sensor was blocked with 1 M ethanolamine. The interaction between rCSE and oxamic hydrazide was measured by injecting increasing concentrations of oxamic hydrazide into the sensor chip. The interaction was measured in 20 mM HEPES (pH 7.4) containing 150 mM NaCl, 0.5 mM TCEP, 100 µM PLP, 0.005% (v/v) Tween-20 and 1% (v/v) DMSO as a cosolvent.

### Preparation of recombinant rCSE

The CSE/pET24a construct was transformed into *E. coli* BL21 cells, which were grown overnight in 1 mL 2 × YT medium in the presence of 20 μg/mL kanamycin (small-scale culture) at 37 °C. The cells were diluted with 150 mL of LB medium containing 20 μg/mL kanamycin and cultured to OD_600_ ~ 0.5–0.8 at 37 °C (large-scale culture). Then, IPTG (final 0.1 mM) was added to the culture medium to induce rCSE and culture was continued for 24 h at 25 °C. Cells were harvested by centrifugation (3000 rpm, 4 °C) for 15 min and resuspended in 2 mL of D-PBS buffer (2.7 mM KCl, 137 mM NaCl, 1.5 mM KH_2_PO_4_ and 8.1 mM Na_2_HPO_4_) supplemented with BugBuster (Novagen, 70,584) and 0.18% protease inhibitor cocktail (Sigma, P8340) on ice for 60 min. The mixture was centrifuged (3000 rpm, 4 °C) for 15 min. The supernatant was purified with a His-tag purification module (Ni Sepharose 6 Fast Flow, GE Healthcare), and desalted with a PD-10 column (GE Healthcare) according to the manufacturer’s instructions. The elution buffer was 50 mM Tris–HCl, 150 mM NaCl, 0.5 mM PMSF, 0.5 mM DTT, 10% glycerol. The collected fractions were analyzed by SDS-PAGE (4–20% acrylamide gradient).

### Preparation of recombinant rCSE for crystal structure determination

*E. coli* BL21 (DE3) cells were transformed with the pET44a vector harboring the rCSE gene and cultured at 37 °C in LB medium to a suitable cell density (OD_600_ 0.4–0.5). Protein expression was induced by adding IPTG (final concentration 0.1 mM). The cells were cultured overnight at 37 °C, then harvested and sonicated in a cell lysis buffer (50 mM Tris–HCl pH 8.0, 500 mM NaCl 1 mM DTT). The expressed rCSE contains a 6 × His-tag and a PreScission protease recognition sequence at the N-terminal. After centrifugation, the proteins were applied to Ni–NTA resin (Qiagen) and eluted with elution buffer (50 mM Tris–HCl pH 8.0, 500 mM NaCl, 500 mM imidazole1, 1 mM DTT). The His-tag moiety was removed by digestion with PreScission protease at 4 °C overnight. The digest was loaded on a HiTrap SP column (GE Healthcare), and rCSE was eluted by applying a gradient of the binding buffer (50 mM Tris–Cl pH 7.0, 1 mM DTT) and the elution buffer (50 mM Tris–Cl pH 7.0, 1 M NaCl, 1 mM DTT). Finally, the proteins were purified by Superdex200pg (GE Healthcare) size exclusion chromatography with crystallization buffer (20 mM Tris–HCl, 200 mM NaCl 1 mM DTT, pH 7.0).

### Crystallization and structure determination of the complex of rCSE with 1

rCSE protein solution was concentrated to ca. 10 mg/mL using Amicon Ultra centrifugal filter units (30 kDa, Merck) and then compound **1**-containing rCSE solution (10 mg/mL rCSE, 1 mM **1**, 20 mM Tris–Cl pH7.0, 200 mM NaCl, 1 mM DTT) was prepared. The complex solution and the reservoir solution (0.18 M tri-ammonium citrate, 20% polyethylene glycol 3350) were mixed in a ratio of 2:1. Crystallization was performed using the sitting drop vapor-diffusion method at 20 °C. The 30% (w/v) glycerol-containing reservoir solution was used as a cryoprotectant to prevent ice formation at 100 K. X-Ray diffraction datasets were collected at beamline BL44XU in SPring-8 (Hyogo, Japan). The collected data were integrated, merged, and scaled with the program XDS^22^ and reduced with Aimless^23^ (Table S1). The initial phases were determined using the program Molrep^24^. After several cycles of manual model building using the program Coot^25^ and structure refinement using the program REFMAC5^26^, electron densities corresponding to **1** and endogenous PLP were clearly identified. They formed a Schiff base in the crystal structure, and a Schiff base linkage model was used for structural refinement. The geometry of the final model was checked by the program PROCHECK^27^ (Table S1) (PDB id: 8J6N).

### Preparation of recombinant rCBS

The rat CBS-HA/pGEX construct was transformed into *E. coli* BL21 cells, which were added to SOC medium containing 20 µg/mL carbenicillin. The cells were grown overnight at 37 °C, diluted with 250 mL of LB medium containing 20 µg/mL carbenicillin and 5-aminolevulinic acid (5-ALA), and cultured at 37 °C overnight. The cells were added to 10 L of LB medium containing carbenicillin and 5-ALA and cultured to OD_600_ = 0.6–0.8 at 37 °C. Then, 0.1 mM IPTG was added and incubation was continued for 24 h at 18 °C. Cells were harvested by centrifugation (5000 G, 4 °C for 5 min), and resuspended in 50 mL of 20 mM HEPES buffer containing 150 mM NaCl, 0.5 mM TCEP and protease inhibitor cocktail. Lysozyme was added on ice for 30 min, then benzonase and triton X-100 (1%) were added and the mixture was sonicated. The sonicate was centrifuged (20,000 G, 4 °C) for 30 min and the supernatant was purified with a GST-tag purification module (GSTrap, GE Healthcare). The elution buffer was HEPES containing 150 mM NaCl, 0.5 mM EDTA, 0.5 mM EGTA and 0.2 mM DTT. The collected fractions were analyzed by SDS-PAGE.

### SDS-PAGE

SDS-PAGE (4–20% polyacrylamide) was performed under denaturing conditions in the presence of 2-mercaptoethanol. Proteins were visualized by staining with Coomassie brilliant blue.

### Bradford assay for protein quantification

Dye reagent was prepared by five-fold dilution of dye reagent (Bio-Rad) with MilliQ water (Merck). A 2 µL aliquot of sample solution was added to 200 µL of the buffer in a 96-well plate. The absorbance was measured at 595 nm. BSA was used as a standard.

### Measurement of fluorescence intensity of HSip-1

The fluorescence intensity of HSip-1 was measured (λ_ex_ = 490 nm, λ_em_ = 510 nm) with a microplate reader (EnVision, Perkin Elmer).

### Cell viability assay

We performed CCK-8 (Cell Counting Kit-8) assay (Dojindo) to examine cell viability. This assay utilizes a highly water-soluble tetrazolium salt, WST-8 (2-(2-methoxy-4-nitrophenyl)-3-(4-nitrophenyl)-5-(2,4-disulfophenyl)-2*H*-tetrazolium monosodium salt), which shows absorbance at ~ 460 nm upon reduction in the presence of electron mediators. This absorbance is proportional to the number of living cells. COS7 cells were plated in flat-bottomed 96-well plate at the density of 1 × 10^4^ cells/mL with 100 µL of DMEM (Gibco 11,885) containing 10% FBS and 1% penicillin streptomycin in a humidified incubator under 5% CO_2_ in air. After incubation for 24 h, 10 µL/well of DMEM containing the indicated concentration of inhibitor was added (0.5% DMSO as a cosolvent) and the cells were further incubated for 28 h. The medium was replaced with 100 µL/well of DMEM containing 10% CCK-8 solution, and the cells were further incubated for 1.5 h. The absorbance at 450 nm was measured with a microplate reader (EnVision, Perkin Elmer).

### Determination of inhibitory activity toward recombinant rCSE by measuring the fluorescence intensity of HSip-1

10 µL of 1.5 mM cysteine solution in 30 mM HEPES (pH 7.4) was added to 10 µL of solution containing 30 µg/mL rCSE, 100 µM PLP, 1 µM HSip-1, 0.005% tween 20 and the test compound (0.5% DMSO as a cosolvent). The mixture was incubated at 25 °C for 3 h and the fluorescence intensity of HSip-1 was measured with a microplate reader.

### Determination of inhibitory activity toward recombinant rCSE by measuring the fluorescence intensity of SSP4

10 µL of 0.5 mM cystine solution in 30 mM HEPES (pH 7.4) was added to 10 µL of solution containing 50 µg/mL rCSE, 100 µM PLP, 1 µM SSP4 (Dojindo), 0.005% tween 20 and various concentrations of the inhibitor (0.5% DMSO as a cosolvent). The mixture was incubated at 37 °C for 60 min and the fluorescence intensity of SSP4 was measured with a microplate reader.

### Transfection of HEK293T cells

Transient transfection of HEK293T cells was performed with Lipofectamine LTX and PLUS reagents (Invitrogen). A mixture of 1 µg plasmid, 1 µL PLUS reagent and 3 µL lipofectamine LTX in 100 µL Opti-MEM (Gibco) was added to the cells (2 mL of the medium).

### Determination of inhibitory activity toward lysate of hCSE-transfected HEK293T cells by measuring the fluorescence intensity of HSip-1

10 µL of 1.5 mM cysteine solution in 30 mM HEPES (pH 7.4) was added to 10 µL of solution containing 300 µg/mL cell lysate of HEK293T cells expressing hCSE, 100 µM PLP, 1 µM HSip-1, 0.005% tween 20 and various concentrations of the inhibitor (0.5% DMSO as a cosolvent). The mixture was incubated at 37 °C for 3 h and the fluorescence intensity of HSip-1 was measured with a microplate reader.

### Determination of inhibitory activity toward recombinant rCBS by measuring the fluorescence intensity of HSip-1

10 µL of 2.5 mM cysteine and 2.5 mM homocysteine in 30 mM HEPES (pH 7.4) was added to 10 µL of a solution containing 20 µg/mL rCBS, 50 µM PLP, 100 µM SAM, 1 µM HSip-1, 0.005% tween 20, 0.5% DMSO and various concentrations of the inhibitors. The mixture was incubated at 37 °C for 2 h and the fluorescence intensity of HSip-1 was measured with a microplate reader.

### Determination of inhibitory activity toward recombinant m3MST by measuring the fluorescence intensity of HSip-1

10 µL of 40 µM DTT and 50 µM 3MP in 30 mM HEPES (pH 7.4) was added to 10 µL of a solution containing 3.8 µg/mL m3MST^16^, 1 µM HSip-1, 0.005% tween 20 and various concentrations of the inhibitor (0.5% DMSO as a cosolvent). The mixture was incubated at 25 °C for 100 min and the fluorescence intensity of HSip-1 was measured with a microplate reader.

### Determination of inhibitory activity toward recombinant MGL by measuring the fluorescence intensity of HSip-1

10 µL of 200 µM homocysteine in 30 mM HEPES (pH 7.4) was added to 10 µL of a solution containing 20 µg/mL MGL (Sigma-Aldrich), 100 µM PLP, 1 µM HSip-1, 0.005% tween 20 and various concentrations of the inhibitor (0.5% DMSO as a cosolvent). The mixture was incubated at 25 °C for 100 min and the fluorescence intensity of HSip-1 was measured with a microplate reader.

### Determination of inhibitory activity toward recombinant ALT by measuring absorbance

An alanine aminotransferase activity assay kit (BioVision, Inc.) was used to measure the activity of ALT. A mixture of L-alanine, NADH, LDH (lactate dehydrogenase) and ALT was incubated at 37 °C for 15 min, then α-ketoglutarate was added to the mixture and the absorbance at 340 nm was measured with a microplate reader.

### Assay of CSE-inhibitory activity of 1 in live cells by fluorescence imaging

COS-7 cells were maintained in Dulbecco’s modified Eagle’s medium (DMEM) containing 10% fetal calf serum (FBS) and 1% penicillin streptomycin in a humidified atmosphere at 37 °C. The cells were seeded in 6 cm dishes at a density of 5 × 10^5^ cells per dish. Transient transfection was performed with Lipofectamine 3000 (Thermo Fisher Scientific). Briefly, 2.5 µg plasmid, 5 µL P3000 reagent, and 7.5 µL Lipofectamine 3000 reagent were mixed in Opti-MEM, and the mixture was added to the cells. After incubation for 24 h, transfected COS-7 cells were re-seeded in eight-well chamber slides (µ-Slide 8 well high, ibidi GmbH) at a density of 4 × 10^4^ cells per well. After incubation for 24 h, the cells were treated with the inhibitor at the indicated concentrations in 10% FBS-DMEM for 24 h. The cells were washed once with serum-free DMEM, followed by incubation with 20 µM SSP4 (Dojindo) in serum-free DMEM containing 500 µM CTAB at 37 °C for 15 min. Excess probe was removed and the cells were washed with HBSS, then fluorescence images were captured using a Nikon A1R + confocal laser scanning microscope with an ECLIPSE Ti-E inverted microscope and a 20 × objective lens. The excitation wavelength was 488 nm and the emission wavelength was 500–550 nm. Fluorescence intensity of images was calculated by using NIS-Elements software (Nikon).

### Quantum chemical calculation and molecular dynamics simulation

Hartree Fock (HF) calculations with the 6-31G* basis set using a polarizable continuum model (PCM) (denoted as HF/6-31G*/PCM) were employed for structural optimization of the complex of **1** and PLP in *cis* and *trans* conformations. Then the conformational energies of the optimized *trans* structure were estimated as the dihedral angle (N–C–C–N) of the dicarbonyl moiety was changed by 10 degree increments. Each structure was optimized by means of HF/6-31G*/PCM calculations with a fixed dihedral angle. All quantum chemical calculations were done by Gaussian 09, Revision C01.

Molecular dynamics (MD) simulations of the complex of ligand and rCSE were performed at constant temperature and pressure (*T* = 10–300 K, *P* = 1 atm) for 10 ns to monitor the state of ligand binding. Chain A in the PDB file was adopted for the initial structure, and water of crystallization was left in the system. The ligand/CSE complex structure was placed at the center of an MD box that extended 12 Å from any solute atom. The MD box was then filled with water molecules to give a water density of 1 g/cm^3^. A Parrinello-Rahman type thermostat and Nosé-Hoover barostat were used to control the system temperature and pressure, respectively. The Amber14SB and generalized amber force field (gaff) were assigned for the protein and the ligand molecule, respectively. Restrained electrostatic potential (RESP) charge estimated by HF/6-31G*/PCM calculation was assigned for the ligand. The TIP3P model was used for solvent water. The cutoff length for van der Waals (vdW) and coulomb interactions in real space was 12 Å. The particle mesh Ewald (PME) method was used for the estimation of the coulomb interactions. The time step for integration of equations of motion was 2 fs. All MD calculations were done by the GROMACS 5.1.4 program.

### Supplementary Information

Supplementary Legends.

Supplementary Information.

Supplementary Movie S1.

## Data Availability

All data relevant to the conclusions of this paper is included in main text, Supplementary Information and Supplementary Movie. All other data are available from the authors upon request.
