# Supplementary material for: Discovery of a cystathionine γ-lyase (CSE) selective inhibitor targeting active-site pyridoxal 5′-phosphate (PLP) via Schiff base formation
Source: Sci Rep. 2023 Sep 30;13:16456. doi: 10.1038/s41598-023-43536-6 (PMC10542788; doi:10.1038/s41598-023-43536-6)
Supplement: Supplementary file 1 — Supplementary Legends. [file 41598_2023_43536_MOESM1_ESM.docx]

**Description of Additional Supplementary File**

File Name: Supplementary Movie S1

Description: **Interconvertibility of the *cis* and *trans* forms of 1 in rCSE by MD calculation at 240 K.** The MD calculation at 240 K was performed using the cocrystal structure of rCSE and **1** determined by the X-ray crystal analysis as shown in Fig. 2b. See also Supplementary Fig. S8.
