# Supplementary material for: Discovery of a cystathionine γ-lyase (CSE) selective inhibitor targeting active-site pyridoxal 5′-phosphate (PLP) via Schiff base formation
Source: Sci Rep. 2023 Sep 30;13:16456. doi: 10.1038/s41598-023-43536-6 (PMC10542788; doi:10.1038/s41598-023-43536-6)
Supplement: Supplementary file 2 — Supplementary Information. [file 41598_2023_43536_MOESM2_ESM.docx]

**Supplementary Information**

Discovery of a Cystathionine *γ*-Lyase (CSE) Selective Inhibitor Targeting

Active-site Pyridoxal 5’-Phosphate (PLP) via Schiff Base Formation

Honami Echizen,^1^ Kenjiro Hanaoka^2,^*, Kazuhito Shimamoto^1^, Ryota Hibi^1^, Sachiko Toma-Fukai^1,3^, Hisashi Ohno^2^, Eita Sasaki^2^, Toru Komatsu^1^, Tasuku Ueno^1^, Yukihiro Tsuchiya^4^, Yasuo Watanabe^4^, Takao Otsuka^5^, Hiroaki Saito^6^, Satoru Nagatoishi^7,8^, Kouhei Tsumoto^7,8^, Hirotatsu Kojima^9^, Takayoshi Okabe^9^, Toshiyuki Shimizu^1^ & Yasuteru Urano^1,10*^

^1^Graduate School of Pharmaceutical Sciences, ^9^Drug Discovery Initiative and ^10^Graduate School of Medicine, The University of Tokyo, 7-3-1 Hongo, Bunkyo-ku, Tokyo 113-0033, Japan. ^2^Graduate School of Pharmaceutical Sciences, Keio University, 1-5-30 Shibakoen, Minato-ku, Tokyo 105-8512, Japan. ^3^Graduate School of Science and Technology, Nara Institute of Science and Technology, Nara 630-0192, Japan. ^4^Faculty of Pharmaceutical Sciences, Showa Pharmaceutical University, Machida-shi 194-8543, Tokyo, Japan. ^5^Graduate School of Medicine, Kyoto University, 53, Shogoin -Kawaharacho, Sakyo-ku, Kyoto 606-8507, Japan. ^6^Faculty of Pharmaceutical Sciences, Hokuriku University, 3 Ho Kanakawa-cho, Kanazawa, Ishikawa 920-1181, Japan. ^7^Medical Device Development and Regulation Research Center, School of Engineering, The University of Tokyo, 7-3-1 Hongo, Bunkyo-ku, Tokyo 113-8656, Japan. ^8^Department of Bioengineering, School of Engineering, The University of Tokyo, 7-3-1, Hongo, Bunkyo-ku, Tokyo 113-8656, Japan.

Correspondence and requests for materials should be addressed to K.H. and Y.U. (K.H., email: khanaoka@keio.jp) (Y.U., email: uranokun@m.u-tokyo.ac.jp).

**Synthesis and characterization of compounds**

Synthesis of HSip-1

HSip-1 was prepared according to the reported method.^SR1^

**Supplementary Figure S1.** Synthesis of oxamic hydrazide (**1**).

Synthesis of 1

Ethyl oxamate (88 mg, 0.75 mmol) and hydrazine monohydrate (75 mg, 1.5 mmol) were dissolved in dry EtOH 4 mL. The solution was stirred at 100℃ for 1 hour in a microwave oven, then cooled to room temperature and washed with EtOH to afford compound **1** as a colorless solid (76.6 mg, 99 % yield) without further purification. ^1^H NMR (400 MHz, DMSO-*d*_6_): δ 4.46 (s, 2H), 7.70 (s, 1H), 7.98 (s, 1H), 9.90 (s, 1H); ^13^C NMR (100 MHz, DMSO-*d*_6_): δ 158.9, 162.4; LRMS [EI^+^]: 103 [M]^+^.

**Supplementary Figure S2.** Synthesis of derivatives of oxamic hydrazide **1**.

Synthesis of 2

Chlorooxoacetic acid ethyl ester (200 mg, 1.4 mmol) and triethylamine (0.22 mL, 1.6 mmol) were dissolved in dry THF (6 mL), and a solution of methylamine (1.6 mmol) in dry THF (6 mL) was added at -20℃. The mixture was stirred at 0℃ for 1 hour, then H_2_O was added to it. The resulting solution was extracted with AcOEt, affording crude **5** as a brown liquid. The extracted brown liquid and hydrazine monohydrate (75 mg, 1.5 mmol) were dissolved in dry EtOH (4 mL). The solution was stirred at 100℃ for 1 hour in a microwave oven, then cooled to room temperature and washed with EtOH to afford compound **2** as a colorless solid (65.2 mg, 38% yield in 2 steps) without further purification. ^1^H NMR (400 MHz, CD_2_Cl_2_) δ 2.84 (d, 3H, *J* = 5.0 Hz), 3.90 (s, 2H), 7.30 (s, 1H), 8.36 (s, 1H); ^13^C NMR (101 MHz, DMSO-*d*_6_) δ 26.2, 158.6, 160.7; LRMS [EI^+^]: 117 [M]^+^.

Synthesis of 3

Chlorooxoacetic acid ethyl ester (100 mg, 0.7 mmol) and triethylamine (0.11 mL, 0.8 mmol) were dissolved in dry THF (3 mL), and a solution of ethylamine (52 μL, 0.8 mmol) in dry THF (2 mL) was added at -20℃. The mixture was stirred at 0℃ for 1 hour, then H_2_O was added to it. The resulting solution was extracted with AcOEt to afford crude **6** as a brown liquid. The extracted brown liquid and hydrazine monohydrate (26 mg, 0.5 mmol) were dissolved in dry EtOH (2 mL). The solution was stirred at 90℃ for 1 hour in a microwave oven, then cooled to room temperature, and washed with EtOH to afford compound **3** as a colorless solid (27.4 mg, 29% yield in 2 steps) without further purification. ^1^H NMR (400 MHz, acetone-*d*_6_): δ 1.14 (t, 3H, *J* = 7.3 Hz), 3.15 (s, 2H), 3.27-3.34 (m, 2H), 8.28 (s, 1H), 10.02 (s, 1H); ^13^C NMR (100 MHz, DMSO-*d*_6_): 15.0, 34.0, 158.8, 160.0; LRMS (EI^+^): 131 [M]^+^.

Synthesis of 4

Chlorooxoacetic acid ethyl ester (100 mg, 0.7 mmol) and triethylamine (0.11 mL, 0.8 mmol) were dissolved in dry THF (3 mL), and a solution of aniline (73 μL, 0.8 mmol) in dry THF (3 mL) was added at -20℃. The mixture was stirred at 0℃ for 1 hour, then H_2_O was added to it. The resulting solution was extracted with AcOEt to afford crude **7** as a brown liquid (69.9 mg). These procedures were repeated twice. The extracted brown liquid (102 mg) and hydrazine monohydrate (53 mg, 1.0 mmol) were dissolved in dry EtOH (4 mL). The mixture was stirred at 90℃ for 1 hour in a microwave oven, then cooled to room temperature, and washed with EtOH to afford compound **4** as a colorless solid (70.5 mg, 36% yield in 2 steps) without further purification. ^1^H NMR (400 MHz, DMSO-*d*_6_): δ 4.61 (s, 2H), 7.11 (t, 1H, *J* = 7.5 Hz), 7.33 (t, 2H, *J* = 8.0 Hz), 7.79 (d, 2H, *J* = 8.0 Hz), 10.25 (s, 1H), 10.54 (s, 1H); ^13^C NMR (100 MHz, DMSO-*d*_6_): δ 120.9, 124.9, 129.2, 138.2, 158.5, 158.9; HRMS (ESI^-^): Calcd for [M - H]^-^, 178.0617, Found, 178.0620 (+0.3 mmu).

**Supplementary Figure S3.** Structures and IC_50_ values (mean ± S.E.) for inhibition of rCSE of 9 commercially available hydrazine and hydrazide compounds. In this assay, 10 µL of 1.5 mM cysteine solution in 30 mM HEPES (pH 7.4) was added to 10 µL of solution containing 30 µg/mL rCSE, 100 µM PLP, 1 µM HSip-1, 0.005 % tween 20 and each compound (3.0, 10, 30 and 100 μM) containing 0.5% DMSO as a cosolvent. The mixture was incubated at 25°C for 3 hours and the fluorescence intensity of HSip-1 was measured with a microplate reader. Although the data of compound **1** was well fitted to a sigmoid plot, NH_2_NH_2_ gave an almost linear plot. The data of NH_2_NHCOCONHNH_2_ also showed an almost linear relationship. A possible explanation is that these compounds bind not only at the active site of rCSE, but also to residues elsewhere in the enzyme. Alternatively, studies over a wider concentration range might yield a sigmoid plot.

**Supplementary Figure S4.** (a) H_2_S production by rCSE in the presence of various concentrations of **1** was measured by gas chromatography. A solution of 30 μg/mL rCSE, 100 μM PLP and 1.5 mM cysteine in 30 mM HEPES buffer (pH 7.4) was incubated at r.t. for 60 min. Error bars represent ±S.D. (n = 4). (b) Inhibitory activity of **1** (mean ± S.E.). H_2_S was detected by gas chromatography. Error bars represent ± S.D. (n = 4).

**Supplementary Figure S5.** Formation of reactive persulfide species by CSE. Cysteine persulfide (Cys-SSH) is formed as the primary product from cystine as a substrate.

**Supplementary Figure S6.** A hydrazide group and an aldehyde group form a stable Schiff base under neutral conditions.

**Supplementary Figure S7.** B-factor plots of compound **1** in *cis* or *trans* form. Hydrogen atoms are not included in the calculation.

There are 12 molecules in an asymmetric unit, and we built all-*cis* or all-*trans* models of the inhibitor and then performed structure refinement. Generally, the B-factor values of neighboring atoms in the same molecule are similar to each other when the electron density of the molecule is clearly visible. Fig. S5 shows the B-factor plots for the all-*cis* or all-*trans* models of the inhibitor. Orange and blue plots indicate the B-factors of the O4 and N3 atoms, respectively. Gray plots show the averaged B-factor values of all other atoms in compound **1** (excluding O4, N3, and hydrogen atoms). The B-factor values calculated after refinement using the all-*cis* model show better concordance than those of the all-*trans* model, which supports the idea that that the *cis* conformation is dominant in the crystal.

**Supplementary Figure S8.** (Continued to the next page)

**Supplementary Figure S8.** (Continued to the next page)

**Supplementary Figure S8.** (Continued to the next page)

**Supplementary Figure S8.** The dihedral angle (N-C-C-N) of the dicarbonyl structure of the PLP-**1** as a function of MD time steps at each indicated temperature. The initial conformation of the ligand was *cis*, and the conformational stability was monitored in the temperature range of 10 K to 300 K.

**Supplementary Figure S9.** Inhibitory activity at 3 µM **1**, **2** and PAG towards RSS-generating enzymes (CSE, CBS and 3MST) and other PLP-dependent enzymes (MGL and ALT). Error bars represent ± S.D. (n = 4).

**Supplementary Figure S10.** Sequence alignment of rCSE and hCSE. The red characters indicate active site amino acid residues involved in recognition of **1** and PLP.

**Supplementary Figure S11.** Viability of COS7 cells in the presence of **1**. COS7 cells were incubated in DMEM (Dulbecco’s modified Eagle’s medium) containing **1** (1, 3, 10, 30 and 100 µM) for 28 hr and the cell viability was measured using CCK-8 reagent. Error bars represent ±S.D. (n = 4).

**Supplementary Figure S12.** Experimental protocol for the indirect assessment of the hCSE-inhibitory activity of **1** in living cells. We firstly prepared hCSE-overexpressing HEK293T cells by transfection and then added various concentrations (10, 30, 100, 300 and 1000 μM) of inhibitor **1** to the cells. After incubation at 37˚C for 30 min, the cells were washed with PBS (two times) and then lysed with RIPA buffer. L-Cysteine (substrate), PLP and HSip-1 were added to the cell lysate (450 μg/mL cell lysate, 1.5 mM L-cysteine, 100 μM PLP, 1 μM HSip-1 in 30 mM HEPES buffer (pH 7.4) containing 0.005% Tween 20), and the fluorescence was measured with a plate reader (Ex. = 490 nm, Em. = 510 nm). The values of % inhibition were calculated according to the equation shown in the figure.

**Supplementary Figure S13.** Volume of the active site of rCSE calculated by UCSF Chimera.

**Table S1. Crystallographic statistics**

|  | *r*CSE |
| --- | --- |
| **Data collectio**n |  |
| X-ray source | SPring-8 BL44XU |
| Space group | C2 |
| Wavelength(Å) | 0.9000Å |
| Cell dimensions |  |
| *a*, *b*, *c* (Å) | 176.2 , 184.1 , 175.9 |
| *α*, *β*, *γ* (°) | 90.0 , 99.5 , 90.0 |
| Resolution (Å) | 48.98-1.90(1.93-1.90)*^a^* |
| *R*_merge_ | 0.107(0.671) |
| *I*/σ*I* | 8.1(2.7) |
| Completeness (%) | 99.2(97.2) |
| Redundancy | 3.8(3.7) |
| **Refinement** |  |
| Resolution (Å) | 49.0-1.9 |
| *R*_work/_ *R*_free_*^b^* (%) | 17.2/19.6 |
| R.m.s deviations |  |
| Bond lengths (Å) | 0.010 |
| Bond angles (º) | 1.584 |
| Ramachandran plots |  |
| Favored | 4545(97.6%) |
| Allowed | 74 (1.6%) |
| Outlier | 36 (0.8%) |

| *^a^* The numbers in parentheses represent statistics in the highest resolution shell. |
| --- |
| *^b^* *R*_free_=∑\|\|*F_o_*\|- \|*F*_c_\|/∑\|*F_o_*\| for 5% of the data not used at any stage of structural refinement. |

**Supplementary Reference:**

SR1) Sasakura, K. et al. Development of a Highly Selective Fluorescence Probe for Hydrogen Sulfide. *J. Am. Chem. Soc.* **133**, 18003–18005 (2011).
